# Cerebrospinal fluid proteomics identifies calcyphosine and follistatin-like 1 as exploratory candidate proteins of interest in hydrocephalus

**DOI:** 10.3389/fneur.2026.1826512

**Published:** 2026-05-29

**Authors:** Hao Han, Xun Xie, Mingchen Xie, Yahui Zhang, Jianhua Cheng, Jian Xu

**Affiliations:** Department of Neurosurgery, The Affiliated Hospital of Qingdao University, Qingdao, China

**Keywords:** calcyphosine, cerebrospinal fluid, follistatin-like 1, hydrocephalus, idiopathic normal pressure hydrocephalus, Olink, post-hemorrhagic hydrocephalus, proteomics

## Abstract

**Background:**

Hydrocephalus comprises etiologically heterogeneous disorders that converge on ventricular enlargement but may be associated with distinct protein-abundance patterns within the cerebrospinal fluid (CSF) compartment. This exploratory study compared CSF proteomic profiles in post-hemorrhagic hydrocephalus (PHH) and idiopathic normal pressure hydrocephalus (iNPH) to characterize CSF protein-abundance patterns associated with these two hydrocephalus and identify proteins for further validation.

**Methods:**

Cerebrospinal fluid samples from 11 participants, including five patients with PHH, three with iNPH, and three non-hydrocephalus controls, were analyzed using Olink proximity extension assay proteomics. Normalized protein expression values were assessed by quality-control analysis, differential expression analysis, and functional annotation using Gene Ontology, KEGG, Reactome, InterPro, Disease Ontology, and STRING-based protein interaction analyses. Differentially expressed proteins were screened using nominal *p* values, with false discovery rate adjustment calculated for statistical interpretation. Calcyphosine (CAPS) and follistatin-like 1 (FSTL1) were further assessed by ELISA in an expanded cohort.

**Results:**

All samples passed quality-control criteria. Compared with the non-hydrocephalus control group, both PHH and iNPH showed predominantly downregulated CSF proteomic profiles, with different exploratory protein-abundance patterns. PHH showed relative CAPS elevation together with reduced proteins related to neuronal structural maintenance, synaptic signaling, axon guidance, immune communication, and extracellular regulation. Functional annotation analyses identified overrepresented terms related to inflammatory signaling, cytokine-receptor interaction, cell adhesion, calcium-related signaling, lysosomal clearance, glycan remodeling, and neural pathways. In iNPH, FSTL1 was relatively increased, whereas proteins involved in synaptic function, axon guidance, cell adhesion, growth-factor signaling, extracellular matrix organization, and cellular stress responses were decreased. Enrichment analyses highlighted neural connectivity, receptor-associated signaling, inflammatory pathways, proteostasis, glycosaminoglycan metabolism, and cilium- or centrosome-related processes. ELISA reproduced the direction of the proteomic findings, showing higher CSF CAPS levels in PHH and higher CSF FSTL1 levels in iNPH than in the non-hydrocephalus control group.

**Discussion:**

The PHH and iNPH share a ventricular phenotype but exhibit distinct CSF proteomic signatures. CAPS and FSTL1 may represent exploratory proteins of interest within different hydrocephalus-related annotation contexts. These findings require validation in larger, independent, longitudinal cohorts before clinical biomarker inferences are made.

## 1 Introduction

Hydrocephalus is a clinical syndrome characterized by pathological enlargement of the ventricular system resulting from disturbed cerebrospinal fluid (CSF) dynamics. Etiologically, hydrocephalus encompasses both secondary and idiopathic forms. Post-hemorrhagic hydrocephalus (PHH) and idiopathic normal pressure hydrocephalus (iNPH) differ substantially in etiology, pathophysiology, and clinical management; however, both impose a considerable neurological burden. The molecular mechanisms underlying these disorders remain incompletely understood, thereby limiting the development of targeted diagnostic and therapeutic strategies (1).

PHH is a severe complication of intracranial hemorrhage, particularly after aneurysmal subarachnoid hemorrhage and intraventricular hemorrhage. Its pathobiology extends far beyond the classical concept of simple mechanical obstruction of CSF circulation. Increasing evidence supports a dynamic, multistage process initiated by erythrocyte lysis and the release of blood-derived toxic mediators, including free hemoglobin and iron (2). These signals rapidly activate microglia and astrocytes, amplify neuroinflammatory cascades, and promote the aberrant expression of profibrotic mediators such as transforming growth factor-β1, thereby contributing to dysregulated choroid plexus secretory and transport functions, subarachnoid fibrosis, and impaired CSF absorption (3). CSF diversion by shunting remains the mainstay of treatment; however, complications such as infection, obstruction, and overdrainage remain frequent, highlighting the limitations of current management strategies (4). At present, no pharmacological therapy has been proven to halt or reverse this process, in large part because the key signaling networks and downstream effector mechanisms have not yet been systematically defined (5).

iNPH is a disorder of uncertain etiology that predominantly affects older adults and is classically characterized by gait disturbance, cognitive impairment, and urinary incontinence in association with ventriculomegaly and a cerebrospinal fluid opening pressure that is usually within the normal range (6). Its pathophysiology is multifactorial and remains incompletely understood. Proposed mechanisms include altered CSF pulsatility and impaired CSF drainage, periventricular white matter injury, chronic cerebral hypoperfusion or hypoxia, disturbed cerebrovascular autoregulation, and reduced parenchymal compliance (7). Chronic low-grade neuroinflammation and abnormalities in CSF biomarker profiles have also been implicated, although published findings remain partly inconsistent and no consensus biomarker signature has been established (8). Shunt surgery can provide meaningful symptomatic benefit in appropriately selected patients; however, a substantial subset shows limited or variable postoperative improvement, and reliable preoperative biomarkers for predicting shunt responsiveness remain lacking (9). The designation “idiopathic” therefore continues to reflect an important gap in our understanding of the upstream molecular events and disease-specific pathways underlying this disorder.

Because CSF is in direct contact with the central nervous system, its proteomic profile provides a dynamic and disease-relevant reflection of the molecular milieu of the brain parenchyma and meningeal interface. Characterizing the CSF proteomic features of PHH and iNPH may therefore improve understanding of their underlying molecular characteristics and pathobiological features.

Traditional mass spectrometry-based proteomic approaches in cerebrospinal fluid studies face several analytical challenges, including interference from high-abundance proteins, limited sensitivity for low-abundance signaling molecules, and the restricted sample volumes typically available in clinical practice. Olink proximity extension assay technology addresses these constraints through paired antibody-based target recognition coupled with DNA reporter amplification, enabling highly sensitive and specific multiplex protein detection from minimal sample input and making it particularly suitable for CSF proteomic profiling (10).

In this exploratory study, we used CSF Olink proximity extension assay proteomics to compare PHH and iNPH with the non-hydrocephalus control group. The aim was to characterize shared and distinct CSF protein-abundance features of these two hydrocephalus subtypes and to identify candidate proteins and enrichment annotations related to inflammation, neural connectivity, extracellular interface remodeling, and CSF microenvironmental imbalance. These findings are intended to provide exploratory biological context and to guide future mechanistic and translational studies in larger cohorts.

## 2 Materials and methods

### 2.1 Participants and CSF sample collection

A total of 11 participants were enrolled from the Affiliated Hospital of Qingdao University and assigned to a PHH group (PHH, *n* = 5; sample IDs THH_01-05), an iNPH group (*n* = 3; sample IDs iNPH_01-03), and a non-hydrocephalus control group (HC, *n* = 3; sample IDs HC_01-03) according to the clinical diagnosis and predefined inclusion criteria. The study was approved by the Ethics Committee of the Affiliated Hospital of Qingdao University (approval no. QYFYWZLL42080), and written informed consent was obtained from all participants or their legal representatives.

Participants were included in the PHH group if they met all of the following criteria: (1) a documented history of intracranial hemorrhage, including aneurysmal subarachnoid hemorrhage, hypertensive intracerebral hemorrhage, traumatic intracranial hemorrhage, intraventricular hemorrhage, or other hemorrhagic events considered by neurosurgeons to be related to subsequent hydrocephalus formation; (2) neuroimaging evidence of ventriculomegaly, characterized by symmetrical enlargement and rounding of the frontal horns of the lateral ventricles; (3) no definite evidence of infection on routine preoperative cerebrospinal fluid and blood testing; and (4) exclusion of congenital ventriculomegaly, intracranial tumors, central nervous system infection, severe post-traumatic structural abnormalities unrelated to the index hemorrhagic event, and compensatory ventricular enlargement secondary to cerebral atrophy. (5) The Evans index > 0.3 (calculated as the ratio of the maximum width of the frontal horns of the lateral ventricles to the maximum intracranial width on the same axial slice).

Participants were included in the iNPH group if they met all of the following criteria: (1) one or more typical clinical features of iNPH, including gait disturbance, urinary dysfunction, and cognitive decline; (2) neuroimaging evidence of ventriculomegaly, characterized by symmetrical enlargement and rounding of the frontal horns of the lateral ventricles; (3) clinical improvement after cerebrospinal fluid drainage testing, particularly in cognition or related neuropsychological function; and (4) absence of subarachnoid hemorrhage, intraventricular hemorrhage, meningitis, significant traumatic brain injury, intracranial tumor, or other identifiable causes of secondary hydrocephalus. Symptomatic improvement after shunt surgery was recorded as supportive diagnostic and follow-up information but was not required for preoperative inclusion. (5) The Evans index > 0.3 (calculated as the ratio of the maximum width of the frontal horns of the lateral ventricles to the maximum intracranial width on the same axial slice).

The non-hydrocephalus control group consisted of patients undergoing elective surgery for primary trigeminal neuralgia or primary hemifacial spasm. These participants were included if they met all of the following criteria: (1) a clinical diagnosis of primary trigeminal neuralgia or primary hemifacial spasm; (2) no neuroimaging evidence of hydrocephalus or ventriculomegaly before surgery; (3) no intracranial hemorrhage, central nervous system infection, intracranial tumor, demyelinating disease, stroke, neurodegenerative disease, or other neurological disorders that could substantially affect CSF composition; (4) no active infection, overt systemic inflammatory state, or malignant tumor at the time of sampling; and (5) no history of CSF shunting or other intracranial interventions that could substantially alter the CSF microenvironment. (6) The Evans index < 0.3 (calculated as the ratio of the maximum width of the frontal horns of the lateral ventricles to the maximum intracranial width on the same axial slice).

All CSF samples were collected intraoperatively. Blood contamination was avoided during sampling. Samples were processed immediately, centrifuged to remove cellular debris, aliquoted, and stored at −80 °C until analysis. Repeated freeze–thaw cycles were avoided.

### 2.2 Olink proteomic profiling and data preprocessing

The CSF samples were profiled using the Olink Reveal whole-proteome panel, which covers approximately 1,034 target proteins. The assay is based on proximity extension assay (PEA) technology with next-generation sequencing readout. Paired oligonucleotide-labeled antibodies bind to adjacent epitopes on the same target protein, allowing oligonucleotide hybridization and extension to generate a unique DNA barcode for downstream quantification. Raw data were quality controlled, normalized, and converted to normalized protein expression (NPX) values. NPX values represent relative protein abundance on a log_2_ scale, and negative values do not indicate technical error.

External controls included sample controls, negative controls, and plate controls. Internal controls included the incubation control, extension control, and amplification control. Total counts per sample were ≥10,000, and assay variation was maintained within an acceptable range (CV < 30%).

The final analytical matrix comprised all 11 study samples and 1,034 target proteins, together with 3 internal technical controls. Plate control normalization was applied, and the analysis report was generated using NPX™ Map version 1.1.3.

### 2.3 Quality control and evaluation of global expression patterns

Quality control followed the standard Olink workflow. QC distribution boxplots, interquartile range plots, Pearson correlation analysis, and unsupervised clustering heatmaps were used to evaluate overall data quality, within-group consistency, between-group separation, and potential outliers. All 11 samples passed sample-level quality control (11/11, 100%), and the overall datapoint pass rate was 100%. Appendix 1 of the Olink Analysis Report was empty, indicating that no assay was excluded because of failure to meet batch release quality control criteria.

The official Olink LOD framework was applied. Missing values, if present, were imputed using the corresponding assay-specific LOD value. No missing values were observed in the final analytical matrix. No target proteins were filtered before differential expression analysis, and all 1,034 target proteins were retained for downstream analysis.

### 2.4 Identification of differentially expressed proteins

Pairwise comparisons were performed between PHH and the non-hydrocephalus control group and between iNPH and the non-hydrocephalus control group using NPX values. Differentially expressed proteins were screened on the basis of nominal *p* values. Benjamini–Hochberg false discovery rate (FDR) correction was also calculated but was not used as the primary criterion for defining differentially expressed proteins. No additional fold-change threshold was imposed. Differential expression results were visualized using volcano plots, Venn diagrams, and heatmaps.

### 2.5 Functional enrichment analyses

Functional enrichment analyses were performed to annotate the differentially expressed protein lists. Gene Ontology (GO) analysis was used to examine overrepresented biological process, cellular component, and molecular function categories. Kyoto Encyclopedia of Genes and Genomes (KEGG) analysis was used to identify overrepresented pathway annotations. Disease Ontology (DO), InterPro, and Reactome databases were used to annotate disease-associated terms, protein-domain categories, and Reactome pathway terms, respectively. All enrichment outputs were interpreted descriptively and in a hypothesis-generating manner rather than as evidence of pathway activation.

### 2.6 Protein–protein interaction network analysis

Protein–protein interaction (PPI) analysis was performed using the STRING database. The resulting network was used to visualize overall connectivity among differentially expressed proteins and to identify proteins occupying relatively central positions within the network. The PPI results were interpreted descriptively and were not considered evidence of direct functional interactions or validated hub proteins.

### 2.7 Statistical considerations

All statistical analyses and visualizations were performed within the standard Olink analytical workflow. Random forest models, receiver operating characteristic curves, and multi-protein diagnostic models were explored during the analytical process. Because of the limited sample size, these results were not presented in the main manuscript. The analytical focus was restricted to differential expression analysis, functional enrichment analysis, and protein–protein interaction network analysis.

### 2.8 Validation of selected proteins by ELISA

The CAPS and FSTL1 were further measured by ELISA using commercially available human kits according to the manufacturers’ instructions. Both assays were based on sandwich ELISA. Patient CSF samples were diluted 2-fold, whereas the non-hydrocephalus control group samples were analyzed without dilution. Absorbance was measured at 450 nm, and standard curves were generated using a four-parameter logistic model. Final concentrations were corrected for the dilution factor. The detection range of the CAPS assay was 0.3125–10 ng/mL, whereas that of the FSTL1 assay was 3.125–100 ng/mL.

The assay layout included standard wells, zero-control wells, blank wells, and sample wells. The ELISA validation cohort comprised CSF samples from 12 patients with PHH, 10 patients with iNPH, and 3 control subjects. Each sample was assayed in triplicate, and the mean value of the three technical replicates was used for subsequent analysis. Detailed raw Olink NPX data, clinical information, and ELISA validation data are provided in the Supplementary material.

## 3 Results

### 3.1 Quality control and global sample distribution characteristics

**Figure 1:**
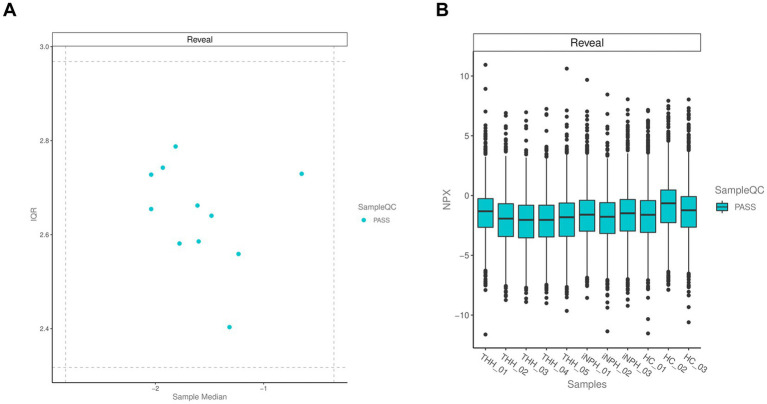
Quality control and global sample distribution characteristics. **(A)** Median-IQR plot for sample-level quality control. **(B)** Boxplots of NPX value distributions across samples.

Because CSF proteomics can be influenced by blood contamination, sample handling, and freeze–thaw cycles, we first performed a systematic quality-control assessment of the NPX data generated by the Olink PEA platform. Global distribution analysis showed that the median NPX values were broadly comparable across PHH, iNPH, and the non-hydrocephalus control group samples, with similar boxplot distributions and only small differences in IQR, indicating no obvious signal drift or abnormal compression (Figure 1). Median-IQR plots further showed that all 11 samples lay within the QC confidence interval, corresponding to an overall pass rate of 100%. Although the PHH samples were obtained months after hemorrhage and may still have contained residual metabolites of prior bleeding, no clear adverse effect on the overall assay performance was observed. These findings indicate that the overall sample quality was reliable and that the dataset was suitable for downstream differential-expression analysis.

### 3.2 CSF proteomic landscape in PHH versus the non-hydrocephalus control group

#### 3.2.1 Overall characteristics of differential expression

**Figure 2:**
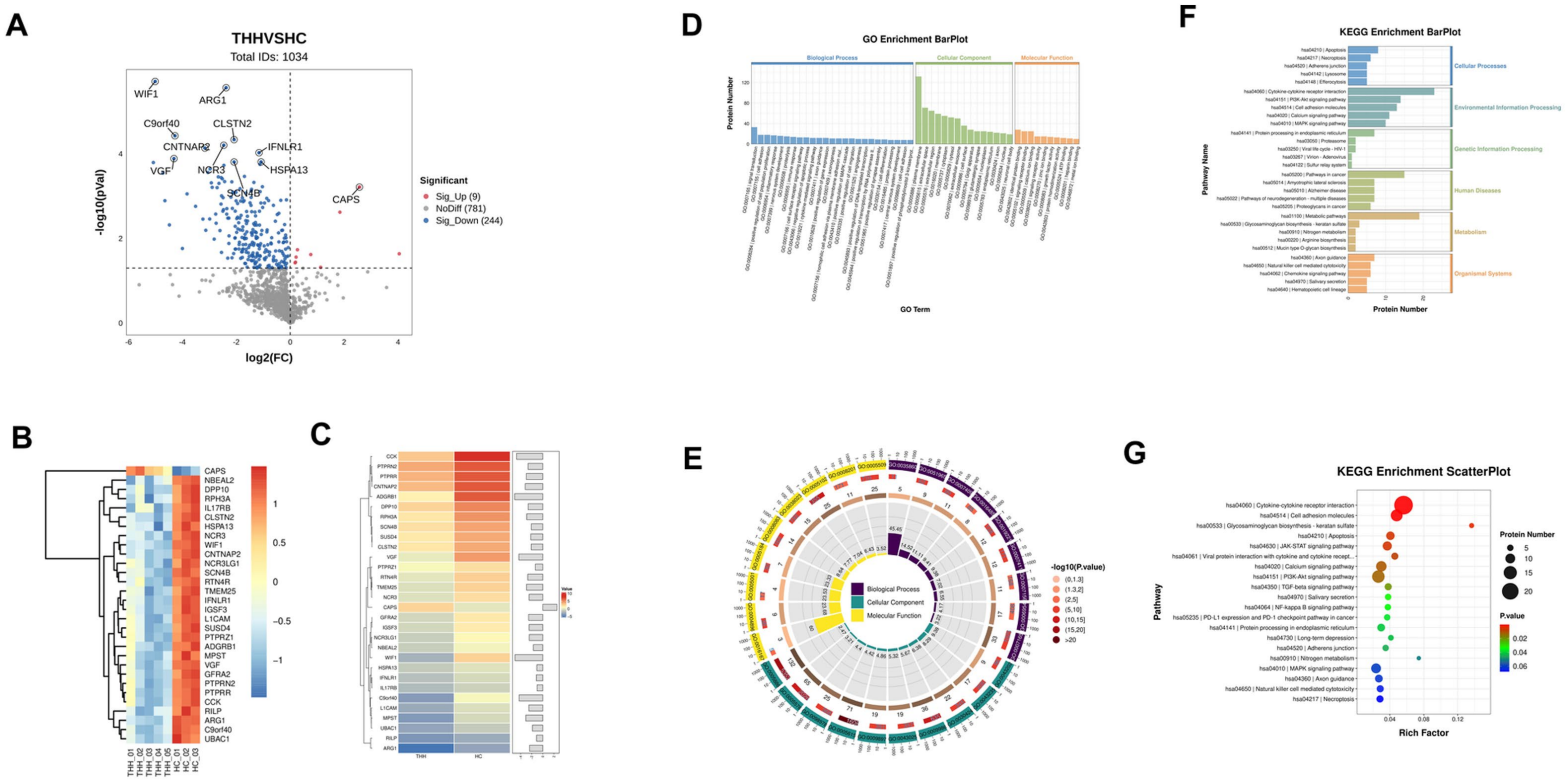
Differential protein landscape and GO/KEGG annotation analysis in PHH versus the non-hydrocephalus control group. **(A)** Volcano plot. **(B)** Heatmap of differentially expressed proteins. **(C)** Group-mean heatmap of differentially expressed proteins. **(D)** GO enrichment bar plot. **(E)** GO circular enrichment plot. **(F)** KEGG enrichment bar plot. **(G)** KEGG enrichment scatter plot.

Compared with the non-hydrocephalus control group, the PHH group showed a CSF proteomic profile dominated by downregulation. In the volcano plot (Figure 2A), significantly downregulated proteins clearly outnumbered upregulated proteins, yielding a left-shifted distribution consistent with a predominance of lower CSF protein abundance in PHH within this dataset. Among these, WIF1 and ARG1 occupied the upper-left portion of the plot and were among the most prominent downregulated proteins by both effect size and statistical significance.

Among the downregulated PHH-associated DEPs, several proteins were annotated to neuronal structural maintenance, synaptic transmission, and axonal plasticity. Proteins involved in cell adhesion, axon guidance, and synaptic connectivity included CNTNAP2, CLSTN2, L1CAM, RTN4R, and ADGRB1. Molecules related to receptor signaling and neurodevelopment included PTPRR, PTPRZ1, GFRA2, IGSF3, and TMEM25. Proteins associated with ion-channel regulation and neuronal excitability included SCN4B and DPP10, whereas proteins linked to vesicle secretion, granule biogenesis, and intracellular trafficking included RPH3A, PTPRN2, and NBEAL2. Neuropeptide or neuromodulatory molecules such as VGF and CCK were also reduced. Overall, this pattern indicates lower relative abundance of CSF proteins annotated to neuronal integrity, synaptic homeostasis, and network plasticity.

Heatmap analysis (Figure 2B) supported this pattern. Most DEPs showed higher expression in the non-hydrocephalus control group and consistently lower expression in PHH, including WIF1, ARG1, CNTNAP2, CLSTN2, NCR3, SCN4B, RTN4R, IFNLR1, IGSF3, L1CAM, PTPRZ1, ADGRB1, MPST, VGF, GFRA2, PTPRN2, PTPRR, CCK, RILP, and UBAC1. By contrast, CAPS (calcyphosine) showed relatively higher expression in PHH and was one of the few proteins moving in the opposite direction. CAPS is an EF-hand calcium-binding protein involved in calcium-related signaling regulation. This trend was also observed in the group-mean heatmap (Figure 2C), where most molecules showed lower average abundance in PHH and relatively higher average abundance in the non-hydrocephalus control group.

In addition to neuronal and synaptic proteins, several immune- and inflammation-related molecules were also decreased, including the innate immune or natural killer cell-associated molecule NCR3 and its ligand NCR3LG1, as well as cytokine receptor-related proteins IL17RB and IFNLR1. These findings indicate lower relative abundance of selected immune-related proteins in PHH CSF. Several proteins involved in metabolic control and intracellular homeostasis were also downregulated, including ARG1, MPST, UBAC1, and RILP, which are related to arginine metabolism, sulfur metabolism and redox regulation, protein ubiquitination, and endosome-lysosome transport, respectively.

Collectively, the PHH-associated DEP list was mainly composed of proteins with lower relative abundance and annotations related to neuronal structural maintenance, synaptic transmission, axonal plasticity, immune communication, and intracellular homeostasis, whereas CAPS was relatively increased. These findings describe an exploratory differential-abundance pattern and should not be interpreted as evidence of a validated PHH molecular phenotype or coordinated pathway-level remodeling.

After adjustment for multiple comparisons using the Benjamini–Hochberg false discovery rate (FDR) procedure, genes with an adjusted *p*-value (*q*-value) < 0.05 were considered statistically significant. The following genes remained significant after FDR correction: WIF1, ARG1, C9orf40, CLSTN2, NCR3, CNTNAP2, IFNLR1, VGF, HSPA13, SCN4B, ADGRB1, RILP, IGSF3, PTPRR, MPST, CCK, NBEAL2, PTPRN2, GFRA2, L1CAM, NCR3LG1, SUSD4, PTPRZ1, TMEM25, DPP10, IL17RB, CAPS, RPH3A, RTN4R, UBAC1, ALK, TNR, NLGN1, PCDH9, PCDHB15, CXADR, GRP, HS6ST2, SLITRK1, SPOCK1, DPP6, PON2, CHST2, IL31RA, TCL1A, CRH, PCDH7, RTBDN, SYT1, BACE1, CTSO, BSG, ISM1, LRRC37A2, CD200, PLXNA4, CPE, TAFA5, POMC, CTSF, NPY, MDGA1, C1QL2, PRKAR1B, PRRT3, TGFA, NPPC, FLT3, POF1B, CCL2, BCAN, NELL1, KIAA0319, TRIM58, CLSTN3, MME, ERN1, IFNAR1, LXN, RSPO3, GFRA3, IFNGR2, CBLN4, and EPHB6.

Given the limited sample size, these FDR-significant findings were interpreted in a hypothesis-generating context and were not intended to represent definitive biological conclusions.

#### 3.2.2 GO annotation profile of the PHH-associated DEP set

The GO annotation and enrichment analysis of DEPs between PHH and the non-hydrocephalus control group (Figures 2D,E) identified overrepresented terms related to extracellular and membrane-associated compartments, receptor binding and signal transduction, inflammatory and immune-related processes, and neurodevelopmental or axon-synapse-associated annotations. These results indicate that the PHH-associated DEP list was distributed across several functional annotation categories, rather than demonstrating coordinated pathway activation.

At the biological process level, the main enriched terms included signal transduction, positive regulation of cell proliferation, inflammatory response, proteolysis, cell surface receptor signaling pathway, cytokine-mediated signaling pathway, axon guidance, negative regulation of apoptotic process, angiogenesis, and positive regulation of phosphatidylinositol 3-kinase signaling. These terms indicate that the PHH-associated DEP set included proteins annotated to extracellular stimulus sensing, receptor-mediated signaling, inflammatory and immune-associated responses, proteolytic activity, and vascular as well as neural structural regulation. The enrichment of nervous system development, axon guidance, and intercellular signaling terms indicates overrepresentation of annotations related to neural network organization and plasticity within the DEP list.

At the cellular component level, DEPs were primarily annotated to the plasma membrane, extracellular region, extracellular region part, membrane raft, cell surface, cytoplasm, extracellular exosome, axon, cytosol, glutamatergic synapse, endoplasmic reticulum lumen, neuronal cell body, and nucleus. This distribution indicates that the PHH-associated DEP set included secreted extracellular proteins, membrane-associated receptors or adhesion-related proteins, and selected neuron-associated structural components. The enrichment of terms such as axon, glutamatergic synapse, and neuronal cell body is consistent with an overrepresentation of proteins linked to neuronal architecture and synaptic organization within the DEP set.

At the molecular function level, enriched terms included identical protein binding, signaling receptor binding, calcium ion binding, signaling receptor activity, cytokine receptor binding, protein homodimerization activity, zinc ion binding, and metal ion binding. These molecular-function terms indicate overrepresentation of ligand-receptor interaction, protein–protein binding, and ion-binding annotations within the PHH-associated DEP set.

Overall, the GO results showed an internally consistent annotation pattern across biological process, cellular component, and molecular function categories. Across these three dimensions, PHH-related DEPs were preferentially mapped to extracellular and membrane-associated compartments and to terms related to receptor binding, signal transduction, inflammatory and immune-associated processes, and neuronal structure-related functions. These findings provide an annotation framework for hypothesis generation regarding PHH-associated CSF proteomic differences, but they should not be taken as mechanistic proof of these processes.

#### 3.2.3 KEGG annotation profile of the PHH-associated DEP set

The KEGG pathway analysis showed that, relative to the non-hydrocephalus control group, DEPs in PHH were enriched across the broad functional categories of environmental information processing, cellular processes, genetic information processing, human diseases, metabolism, and organismal systems (Figures 2F,G). At a descriptive level, the PHH-associated DEP set mapped to pathway annotations related to immune-inflammatory signaling, cell death and clearance, cell adhesion and junctional regulation, proteostasis-associated stress responses, glycan and metabolic remodeling, and neural signaling.

Among the most prominently enriched pathways were cytokine-cytokine receptor interaction and several other immune-inflammatory pathways, including the JAK–STAT signaling pathway, NF-κB signaling pathway, chemokine signaling pathway, and natural killer cell-mediated cytotoxicity. These findings indicate that proteins in the PHH-associated DEP set mapped to inflammatory and immune-regulatory signaling annotations. Enrichment of the PD-L1 expression and PD-1 checkpoint pathway in cancer may likewise indicate representation of proteins involved in broader immunoregulatory circuitry.

Enrichment of cell adhesion molecules and adherens junction pathways indicates overrepresentation of proteins annotated to cell–cell adhesion, intercellular junctional organization, and barrier-related molecular interfaces. Similarly, enrichment of glycosaminoglycan biosynthesis – keratan sulfate and mucin-type O-glycan biosynthesis indicates overrepresentation of glycan-related extracellular interface annotations.

Annotation terms related to cellular injury and clearance, including apoptosis, necroptosis, lysosome, and efferocytosis, were also enriched, indicating that the PHH-associated DEP set contained proteins mapped to cell-damage, regulated cell-death, and phagocytic-clearance categories. At the same time, enrichment of the PI3K-Akt signaling pathway, MAPK signaling pathway, calcium signaling pathway, and TGF-β signaling pathway indicates representation of proteins annotated to stress-responsive signaling, cell-survival-related categories, and repair-associated processes.

In addition, enrichment of protein processing in the endoplasmic reticulum and proteasome pathways indicates annotation to protein folding and degradation processes, whereas enrichment of metabolic pathways, nitrogen metabolism, and arginine biosynthesis indicates broader metabolic pathway representation within the DEP set. The identification of neural system-related pathways, such as axon guidance and long-term depression, further indicates that proteins annotated to neural connectivity and synaptic plasticity were present among the enriched categories. Collectively, these KEGG results provide an exploratory pathway-annotation framework for the PHH-associated DEP set and identify candidate biological themes for subsequent validation, rather than definitive evidence for specific pathogenic mechanisms.

#### 3.2.4 Integrative DO, InterPro, Reactome annotation and protein covariation patterns in PHH

**Figure 3:**
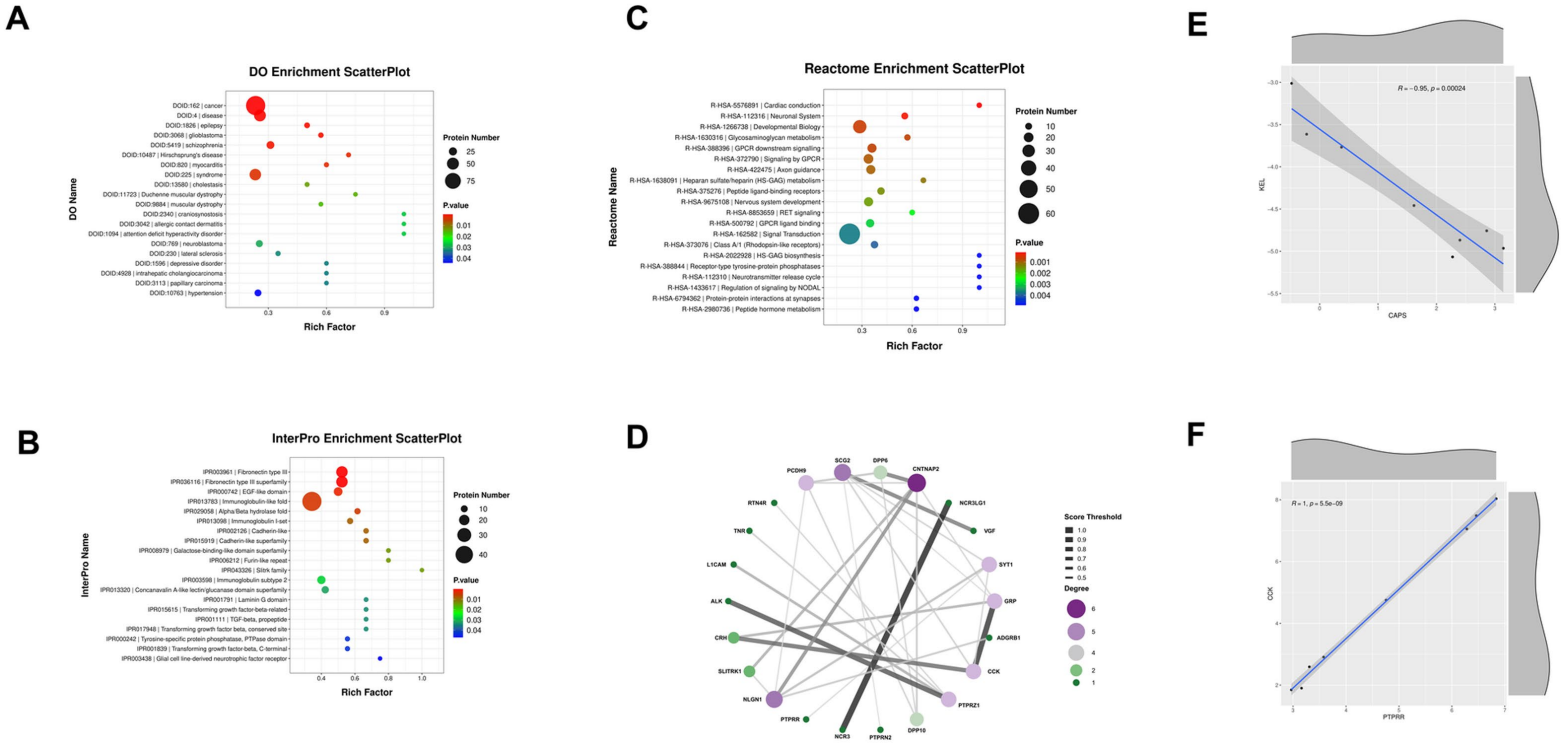
Reactome/InterPro/DO annotation analyses and protein–protein interaction network analysis of differentially expressed proteins in PHH versus the non-hydrocephalus control group. **(A)** Disease Ontology enrichment scatter plot. **(B)** InterPro enrichment scatter plot. **(C)** Reactome enrichment scatter plot. **(D)** STRING-based protein–protein interaction network. **(E)** Correlation analysis between KEL and CAPS. **(F)** Correlation analysis between CCK and PTPRR.

Using the DEPs identified between PHH and the non-hydrocephalus control group by Olink, we conducted an integrative analysis of Disease Ontology (DO) annotation, protein domain enrichment, Reactome pathway enrichment, and protein covariation patterns. DO enrichment (Figure 3A) yielded broad parent terms such as disease and syndrome, together with more specific annotations related to neoplastic conditions, neuropsychiatric or neurodevelopmental disorders, and inflammatory or organ-system-related processes. These annotations should not be interpreted as indicating the presence of those specific diseases in PHH. Rather, they indicate that PHH-associated DEPs partially overlap with annotation terms shared across multiple pathological contexts, particularly those related to cell adhesion, developmental regulation, inflammatory signaling, and tissue remodeling.

InterPro enrichment (Figure 3B) provided complementary protein-domain annotation. Significantly enriched terms included fibronectin type III and the fibronectin type III superfamily, immunoglobulin-like fold and I-set, cadherin-like and the cadherin-like superfamily, EGF-like domain, laminin G domain, and furin-like repeat. Protein tyrosine phosphatase domains, TGF-β superfamily-related domains, and glial cell line-derived neurotrophic factor receptor-related terms were also enriched. Collectively, these domain-level features indicate representation of proteins annotated to cell–cell and cell-matrix interactions, receptor-ligand communication, proteolytic processing of secreted proteins, and neurodevelopmental or neurotrophic signaling.

Reactome analysis (Figure 3C) further grouped the enriched terms into three broad functional categories. The first was enriched for nervous system- and synapse-related pathways, including Neuronal System, Neurotransmitter release cycle, protein–protein interactions at synapses, Axon guidance, and Nervous system development. The second was enriched for receptor-mediated signaling pathways, including GPCR ligand binding, signaling by GPCR, GPCR downstream signaling, Class A/1 (Rhodopsin-like receptors), Signal Transduction, Receptor-type tyrosine-protein phosphatases, RET signaling, and Regulation of signaling by NODAL. The third comprised glycosaminoglycan- and heparan sulfate-related pathways, including Glycosaminoglycan metabolism, Heparan sulfate/heparin metabolism, and HS-GAG biosynthesis. Additional enrichment of Peptide hormone metabolism and Cardiac conduction may represent annotation overlap with neuroendocrine-like signaling and excitability-associated categories within the dataset. The Reactome profile showed overrepresentation of terms related to neural signaling, cell-surface receptor pathways, and extracellular glycan or matrix-associated biology in PHH.

At the level of STRING-based protein interaction and dataset-level covariation (Figures 3D–F), the PHH-versus-non-hydrocephalus control group DEPs formed a relatively sparse interaction network containing several proteins with related annotations. In the circular PPI visualization (Figure 3D), CNTNAP2 displayed the highest connectivity among the included nodes, with other relatively connected proteins including NLGN1, SCG2, PCDH9, SYT1, GRP, CCK, and PTPRZ1. The network was mainly composed of neural adhesion- or connectivity-related molecules, including CNTNAP2, NLGN1, PCDH9, SLITRK1, L1CAM, TNR, and RTN4R; synaptic or neuropeptide-associated proteins, including SYT1, VGF, CCK, GRP, CRH, SCG2, DPP6, and DPP10; and receptor- or signaling-related molecules, including PTPRZ1, PTPRR, ALK, ADGRB1, NCR3, and NCR3LG1.

In the correlation analyses (Figures 3E,F), PTPRR and CCK showed a numerically perfect positive correlation within the present dataset (*R* = 1, *p* = 5.5 × 10^−9^), whereas CAPS and KEL showed a strong inverse correlation (*R* = −0.95, *p* = 0.00024). Given the limited sample size, these findings should not be interpreted as evidence of validated hub proteins or direct mechanistic dependencies. Rather, they are best regarded as exploratory dataset-level features, indicating covariance patterns among proteins annotated to neural adhesion, synaptic/neuropeptide signaling, and receptor-associated categories in the CSF compartment.

### 3.3 CSF proteomic landscape in iNPH versus the non-hydrocephalus control group

#### 3.3.1 Overall characteristics of differential expression

**Figure 4:**
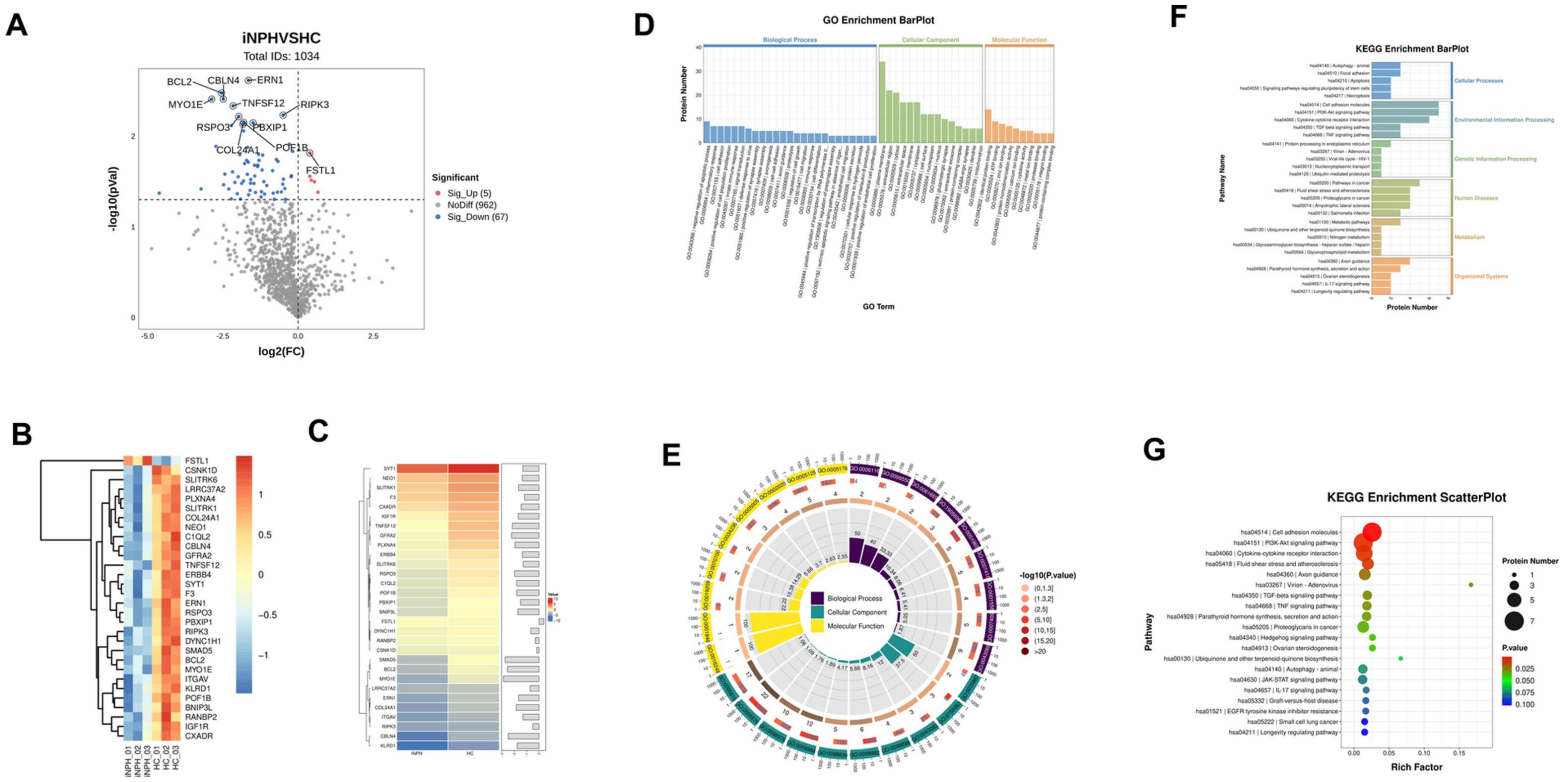
Differential protein landscape and GO/KEGG annotation analysis in iNPH versus the non-hydrocephalus control group. **(A)** Volcano plot. **(B)** Heatmap of differentially expressed proteins. **(C)** Group-mean heatmap of differentially expressed proteins. **(D)** GO enrichment bar plot. **(E)** GO circular enrichment plot. **(F)** KEGG enrichment bar plot. **(G)** KEGG enrichment scatter plot.

Compared with the non-hydrocephalus control group, the iNPH group also exhibited a CSF proteomic profile characterized predominantly by downregulation. In the volcano plot (Figure 4A), proteins meeting the significance threshold were distributed mainly within the negative log2(FC) region, with significantly downregulated proteins markedly outnumbering upregulated proteins, resulting in an overall left-shifted pattern. This distribution is consistent with lower CSF abundance of many proteins in iNPH relative to the non-hydrocephalus control group within this dataset. Among the limited number of relatively increased proteins, FSTL1 was positioned prominently on the right-hand side of the plot. As a secreted glycoprotein previously associated with tissue remodeling, angiogenesis, and immune regulation, the relative increase in FSTL1 may represent an exploratory signal related to microenvironment-associated remodeling in iNPH.

The downregulated iNPH-associated DEPs included many proteins annotated to neural connectivity, synaptic transmission, and axon-guidance-associated signaling. SYT1 is closely linked to synaptic vesicle exocytosis and neurotransmitter release. SLITRK1 and SLITRK6 are involved in neurite outgrowth and synapse formation or maintenance. PLXNA4 participates in semaphorin–plexin-mediated axon-guidance signaling. NEO1 is associated with cell adhesion and developmental signaling, whereas C1QL2 and CBLN4 are more appropriately regarded as synaptic organizing molecules involved in trans-synaptic communication. GFRA2, IGF1R, and ERBB4 represent neurotrophic or receptor tyrosine kinase-related signaling axes, and RSPO3 functions as a potentiator of Wnt signaling. Collectively, the reduced abundance of these proteins indicates lower relative abundance of CSF proteins annotated to neural network maintenance, synaptic homeostasis, and developmental support in iNPH.

Heatmap analyses further supported this overall pattern. At the individual-sample level (Figure 4B), multiple DEPs showed relatively higher expression in the non-hydrocephalus control group and lower expression in iNPH, including SYT1, NEO1, SLITRK1, GFRA2, PLXNA4, ERBB4, C1QL2, IGF1R, and CXADR. The group-mean heatmap (Figure 4C) demonstrated the same directional trend, with lower average expression of most DEPs in iNPH than in the non-hydrocephalus control group, indicating a consistent group-level expression pattern. CXADR is more appropriately interpreted as a molecule related to cell adhesion and tight-junction integrity; in the present dataset, its lower abundance should be viewed as an exploratory observation involving an interface-associated protein. In contrast, FSTL1 was among the few proteins showing a relative increase.

In addition to neural and synaptic proteins, several molecules related to extracellular matrix organization, cell adhesion, and microenvironmental homeostasis were also downregulated. COL24A1 is a member of the collagen family. ITGAV encodes integrin alphaV and participates in cell-matrix adhesion and recognition of multiple extracellular ligands. F3 encodes tissue factor and is more appropriately considered a molecule located at the interface of coagulation and vascular-inflammatory signaling. TNFSF12 (TWEAK), a member of the TNF ligand family, is involved in inflammatory regulation and tissue remodeling. These observations indicate that the iNPH-associated DEP list included proteins annotated not only to neural network-related categories, but also to extracellular matrix organization, barrier-associated interfaces, and local inflammatory or vascular response categories.

Several proteins associated with stress responses, cell-fate regulation, and intracellular homeostasis were likewise reduced. ERN1 is a key sensor of the endoplasmic reticulum unfolded protein response. RIPK3 is a core kinase involved in regulated necrosis. BCL2 regulates the balance between cell survival and apoptosis. BNIP3L (NIX) is more appropriately classified as a molecule involved in mitophagy and cell-fate control, whereas SMAD5 participates in TGF-*β*/BMP signaling. Additional decreases were observed in DYNC1H1, MYO1E, RANBP2, and PBXIP1, which are related to microtubule-dependent retrograde transport, actin-based motor function, nucleocytoplasmic transport and SUMOylation, and transcription-related protein interactions, respectively. Together, these findings indicate that the iNPH-associated DEP list included proteins annotated not only to neural connectivity and synaptic function but also to stress adaptation, cellular homeostasis, and subcellular organization. The immune-related receptor KLRD1 also showed lower relative abundance, adding to the representation of immune-associated annotations within the dataset.

Overall, compared with the non-hydrocephalus control group, the iNPH-associated DEP list was predominantly composed of proteins with lower relative abundance and annotations related to neural connectivity and synaptic transmission, axon-guidance and developmental signaling, growth factor receptor- and Wnt-associated categories, extracellular matrix and microenvironmental homeostasis, and stress-response and cell-fate regulation. In contrast, only a limited number of proteins, including FSTL1, showed relative increases. This differential-abundance profile should be interpreted as an exploratory description of the present dataset rather than as evidence of coordinated iNPH molecular programs.

Notably, in the iNPH group (*n* = 3), no proteins remained statistically significant after Benjamini-Hochberg false discovery rate (FDR) correction (all *q* > 0.05). Given the limited sample size, these findings were interpreted in a hypothesis-generating context and were not intended to represent definitive biological conclusions.

#### 3.3.2 GO annotation profile of the iNPH-associated DEP set

The GO annotation and enrichment analysis of the DEPs identified in iNPH versus the non-hydrocephalus control group (Figures 4D,E) showed enrichment in extracellular and membrane-associated compartments, receptor-binding and ion-binding molecular functions, and biological processes related to inflammatory response, signal transduction, regulation of proliferation or apoptosis, cell adhesion, and neural connectivity. Collectively, these findings indicate that the iNPH-associated DEP list was annotated across multiple functional categories rather than being restricted to a single pathway category, with enrichment patterns involving extracellular communication, local microenvironmental regulation, and neural structure-related processes.

At the biological process level, the principal enriched terms included negative regulation of apoptotic process, inflammatory response, signal transduction, positive regulation of cell proliferation, and regulation of cell adhesion. Terms related to synapse assembly, axon guidance, positive regulation of cell growth, and transcriptional regulation were also enriched. These results indicate that iNPH-associated DEPs included proteins annotated to inflammatory and receptor-mediated signaling processes, cell-survival control, cell–cell adhesion, and neural connectivity-related categories. In particular, enrichment of synapse assembly and axon guidance indicates overrepresentation of annotations related to neuronal network organization and connectivity within the DEP list.

At the cellular component level, DEPs were mapped predominantly to extracellular exosome, plasma membrane, extracellular region, extracellular space, cell surface, cytoplasm, cytosol, and nucleus, together with neuron-related structures such as glutamatergic synapse and neuronal cell body. This distribution indicates that the altered CSF proteins in iNPH are composed largely of secreted extracellular proteins, membrane-associated components, and selected intracellular proteins, with additional representation of neuron-related structural elements. Enrichment of glutamatergic synapse and neuronal cell body likewise indicates overrepresentation of neuron-related structural annotations within the iNPH-associated DEP set.

At the molecular function level, the dominant enriched terms included calcium ion binding, signaling receptor binding, several receptor-associated binding categories, protein homodimerization activity, integrin binding, and protein-containing complex binding. These molecular-function results indicate enrichment of ligand-receptor interaction, ion-binding, and protein-complex assembly annotations, with related terms involving extracellular signal recognition, receptor-mediated communication, and adhesion- or complex-associated regulation within the iNPH-associated DEP set.

The GO enrichment results were directionally concordant across biological process, cellular component, and molecular function categories. The DEPs identified in iNPH were mainly mapped to extracellular and membrane-associated compartments and to annotations related to inflammatory response, signal transduction, cell adhesion, regulation of cell survival, and neural connectivity-related processes. These findings support an exploratory interpretation in which the iNPH-associated DEP list shows overrepresentation of annotations related to extracellular signaling, local microenvironmental regulation, and neural structure-related categories.

#### 3.3.3 KEGG annotation profile of the iNPH-associated DEP set

The KEGG pathway enrichment analysis based on the differential CSF proteins (Figures 4F,G) showed that the principal enriched pathways in iNPH relative to the non-hydrocephalus control group were distributed across six broad functional modules: Cellular Processes, Environmental Information Processing, Genetic Information Processing, Human Diseases, Metabolism, and Organismal Systems. At an overall level, these enrichment patterns indicate that the iNPH-associated DEP list mapped to multiple annotation domains, including cell adhesion and signaling, inflammatory cytokine-related pathways, cellular stress and death categories, proteostasis, metabolic regulation, and neural signaling.

Among the more highly represented pathways were Cell adhesion molecules and the PI3K-Akt signaling pathway, both of which involved a relatively large number of proteins. Cytokine-cytokine receptor interaction was also prominently enriched, together with the TGF-β signaling pathway and TNF signaling pathway. In the scatter plot, IL-17 signaling pathway and JAK–STAT signaling pathway were likewise among the leading entries. Collectively, these findings indicate enrichment of pathway annotations related to cell-adhesion-associated interactions and inflammatory signaling within the iNPH CSF proteomic dataset. The appearance of Fluid shear stress and atherosclerosis further indicates that a subset of DEPs mapped to pathway categories involving vascular mechanotransduction and inflammation-associated adhesion signaling.

Within the Cellular Processes module, Autophagy – animal, Focal adhesion, Apoptosis, and Necroptosis were enriched, indicating that the iNPH-associated DEP set included proteins mapped to pathway categories related to stress adaptation, adhesion-associated signaling, mechanotransduction, and regulated cell-death processes. Enrichment of Signaling pathways regulating pluripotency of stem cells additionally indicates that some proteins in the dataset were annotated to cell-state regulation and repair-associated signaling categories.

Within Genetic Information Processing, Protein processing in endoplasmic reticulum, Ubiquitin mediated proteolysis, and Nucleocytoplasmic transport were enriched, indicating representation of pathways related to protein folding, processing, degradation, and intracellular trafficking. Terms such as Virion – Adenovirus and Viral life cycle – HIV-1 also appeared in the enrichment output; however, these are more appropriately interpreted as reflecting annotation overlap with host inflammatory, processing, and transport pathways rather than evidence of specific viral infection.

Within the Human Diseases module, Pathways in cancer, Proteoglycans in cancer, Fluid shear stress and atherosclerosis, and Amyotrophic lateral sclerosis were among the leading entries. These terms are best interpreted as reflecting enrichment of shared molecular modules related to cell adhesion, proteoglycan-associated extracellular remodeling, stress signaling, and neural dysfunction, rather than indicating the presence of the corresponding diseases.

Within metabolism, the broad category of metabolic pathways was enriched together with Ubiquinone and other terpenoid-quinone biosynthesis, Nitrogen metabolism, Glycerophospholipid metabolism, and Glycosaminoglycan biosynthesis-heparan sulfate/heparin. These findings indicate representation of energy-related, membrane lipid metabolism, nitrogen-related, and glycosaminoglycan-associated pathway annotations. The enrichment of glycosaminoglycan-related pathways is also directionally concordant with the appearance of Proteoglycans in cancer and Cell adhesion molecules and supports annotation-level overrepresentation of extracellular interface- and matrix-associated categories.

Within Organismal Systems, Axon guidance was among the higher-coverage pathways, and the scatter plot also included Parathyroid hormone synthesis, secretion and action, Ovarian steroidogenesis, and the Longevity regulating pathway. These results indicate that, in addition to inflammatory and stress-response-related pathway annotations, the iNPH proteomic dataset also included pathway categories linked to neural connectivity and broader organism-level regulatory signaling. Collectively, the KEGG profile supports an exploratory interpretation in which the iNPH-associated DEP list cannot be reduced to a single pathway category and instead showed enrichment of annotations related to extracellular signaling, cellular stress responses, metabolism, and neural structural regulation.

#### 3.3.4 Integrative DO, InterPro, Reactome annotation and protein covariation patterns in iNPH

**Figure 5:**
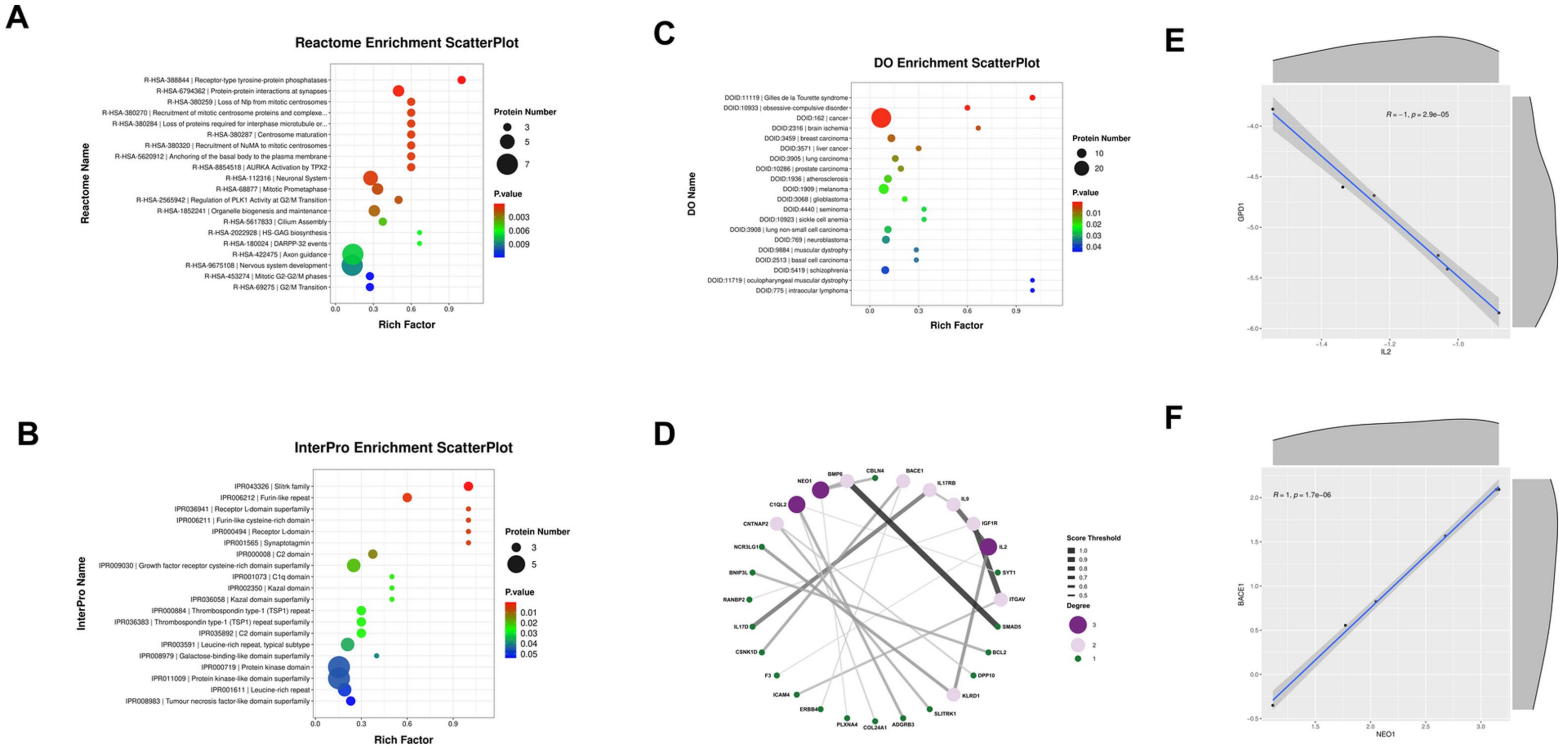
Reactome/InterPro/DO annotation analyses and protein–protein interaction network analysis of differentially expressed proteins in iNPH versus the non-hydrocephalus control group. **(A)** Reactome enrichment scatter plot. **(B)** InterPro enrichment scatter plot. **(C)** Disease Ontology enrichment scatter plot. **(D)** STRING-based protein–protein interaction network. **(E)** Correlation analysis between IL2 and GPD1. **(F)** Correlation analysis between NEO1 and BACE1.

Using the DEPs identified between iNPH and HC by Olink, we performed an integrative analysis of Disease Ontology (DO) annotation, protein domain enrichment, Reactome pathway enrichment, and protein covariation patterns. DO enrichment (Figure 5C) was dominated by tumor-related annotations among the top significant terms, including cancer, breast carcinoma, liver cancer, lung carcinoma, prostate carcinoma, melanoma, glioblastoma, seminoma, basal cell carcinoma, and intraocular lymphoma. A smaller number of neuropsychiatric and vascular or ischemia-related terms were also identified, including Gilles de la Tourette syndrome, obsessive-compulsive disorder, schizophrenia, brain ischemia, and atherosclerosis. Rather, they indicate that the iNPH-associated DEPs partially overlap with annotation terms shared across multiple pathological contexts, particularly those related to neural dysfunction, vascular-associated processes, and extracellular remodeling.

InterPro enrichment (Figure 5B) provided complementary protein-domain annotation. Enriched domains included the Slitrk family, Synaptotagmin, C2 domain, and C2 domain superfamily, together with Receptor L-domain, Receptor L-domain superfamily, Furin-like repeat, Furin-like cysteine-rich domain, and growth factor receptor cysteine-rich domain superfamily. Domains related to complement activity, proteolysis, and extracellular matrix-associated biology were also represented, including C1q domain, Kazal domain, Thrombospondin type-1 repeat, galactose-binding-like domain superfamily, leucine-rich repeat, protein kinase domain, and tumor necrosis factor-like domain superfamily. Collectively, these domain-level features indicate representation of proteins annotated to neural connectivity, calcium-dependent vesicle-associated secretion, receptor-ligand recognition, extracellular interface structure, and immune- or matrix-related homeostatic processes.

Reactome analysis (Figure 5A) further organized the enriched terms into several broad functional categories. One category involved receptor-associated and synapse-related pathways, including Receptor-type tyrosine-protein phosphatases and Protein–protein interactions at synapses. A second category involved nervous system development and neural connectivity, including Neuronal System, Axon guidance, Nervous system development, and DARPP-32 events. A third category comprised cilium-, basal body-, and centrosome-associated processes, including Cilium Assembly, Anchoring of the basal body to the plasma membrane, Centrosome maturation, and Recruitment of NuMA to mitotic centrosomes. A fourth category included cell-cycle- and mitosis-related pathways, such as AURKA activation by TPX2, Regulation of PLK1 activity at G2/M transition, Mitotic Prometaphase, Mitotic G2-G2/M phases, and G2/M Transition. Additional enrichment of HS-GAG biosynthesis indicates representation of glycosaminoglycan-related pathway annotations within the iNPH-associated DEP set. Overall, the Reactome profile showed overrepresentation of terms related to neural signaling, receptor-associated pathways, cilium- or centrosome-related biology, and extracellular glycan-associated processes in iNPH.

At the level of STRING-based protein interaction and dataset-level covariation (Figures 5D–F), the iNPH-versus-non-hydrocephalus control group DEPs formed a relatively sparse interaction network that was interpreted descriptively. In the circular PPI visualization (Figure 5D), IL2, C1QL2, and NEO1 displayed the highest connectivity among the included nodes, whereas BACE1, BMP6, CNTNAP2, IL17RB, IL9, IGF1R, ITGAV, and KLRD1 showed intermediate connectivity. The network comprised several proteins related to neural connectivity or synaptic function, including C1QL2, NEO1, CNTNAP2, SYT1, SLITRK1, CBLN4, DPP10, PLXNA4, and ADGRB3; inflammatory or immune-associated molecules, including IL2, IL9, IL17RB, IL17D, KLRD1, NCR3LG1, and ICAM4; and signaling or extracellular interface-related proteins, including IGF1R, BMP6, SMAD5, ITGAV, F3, COL24A1, ERBB4, and BACE1.

In the correlation analyses (Figures 5E,F), NEO1 and BACE1 showed a numerically perfect positive correlation within the present dataset (*R* = 1, *p* = 1.7 × 10^−6^), whereas IL2 and GPD1 showed a numerically perfect negative correlation (*R* = −1, *p* = 2.9 × 10^−5^). Given the limited sample size, these findings should not be interpreted as evidence of validated hub proteins or direct mechanistic dependencies. Rather, they are better regarded as exploratory dataset-level features, indicating covariance patterns among proteins annotated to neural connectivity-related, inflammatory or immune-associated, and extracellular interface- or signaling-related categories in the CSF compartment.

Collectively, relative to the non-hydrocephalus control group, the iNPH-associated differential-abundance profile showed enrichment patterns involving neural connectivity, synapse- and axon guidance-related annotations, receptor-associated extracellular domains and ligand-recognition modules, cilium- or centrosome-related categories, and HS-GAG biosynthesis or extracellular interface-associated homeostasis. These findings support an exploratory interpretation in which the iNPH-associated DEP list showed enrichment across several functionally related annotation categories rather than a single isolated pathway category.

### 3.4 ELISA validation of CAPS and FSTL1 in the expanded cohort

Given the limited sample size of the Olink proteomic discovery cohort, ELISA was subsequently performed in an expanded validation cohort to provide orthogonal support for the initial screening results and to assess whether the observed differential expression trends could be reproduced in a larger patient sample set. The validation cohort comprised 12 patients with PHH, 10 patients with iNPH, and 3 control subjects. During the assay, patient CSF samples were measured after 2-fold dilution, whereas control samples were analyzed without dilution. Final concentrations were calculated from the standard curve and corrected for the corresponding dilution factor. Each CSF sample was assayed in technical triplicate, and the mean concentration of the three replicate wells was used for statistical analysis.

**Figure 6:**
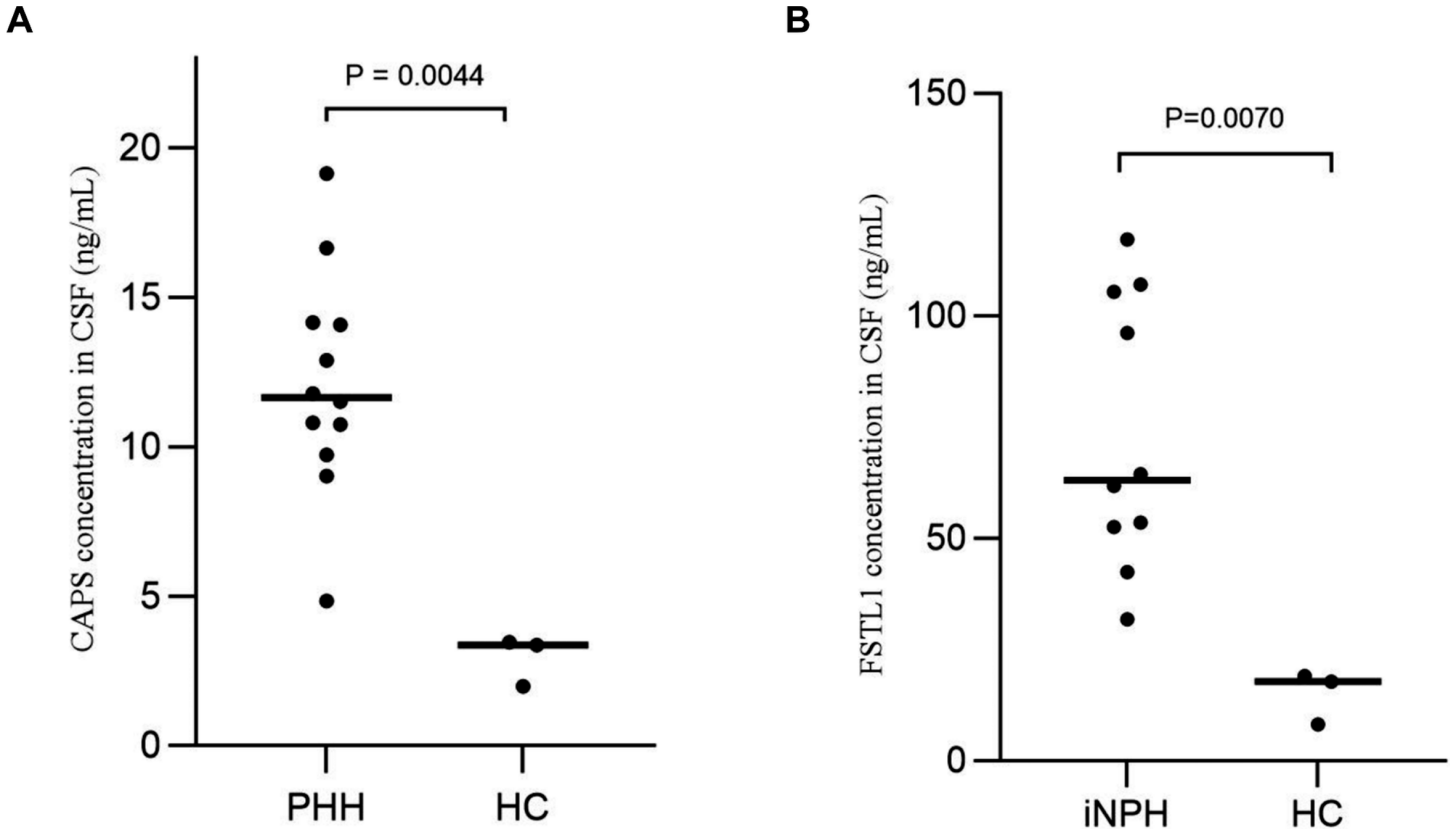
ELISA validation of CAPS and FSTL1 in the expanded cohort. **(A)** CSF CAPS levels in PHH and the non-hydrocephalus control group. **(B)** CSF FSTL1 levels in iNPH and the non-hydrocephalus control group.

The ELISA validation (Figure 6A) showed that CSF CAPS levels were significantly higher in the PHH group than in controls. The median CAPS concentration was 11.64 ng/mL in PHH samples and 3.37 ng/mL in control samples, with a significant between-group difference by two-tailed Mann–Whitney U test (*p* = 0.0044). This result was consistent with the direction of change observed in the Olink proteomic analysis. Similarly, CSF FSTL1 levels (Figure 6B) were significantly elevated in the iNPH group compared with controls. The median FSTL1 concentration was 63.12 ng/mL in iNPH samples and 17.78 ng/mL in control samples, and the difference was also significant by two-tailed Mann–Whitney U test (*p* = 0.0070). These findings indicate that the increased CAPS signal in PHH and the increased FSTL1 signal in iNPH were reproduced in the ELISA validation cohort, supporting the consistency of the candidate-protein trends identified in the discovery analysis.

Importantly, this supplementary validation was intended to strengthen the reproducibility of the Olink-based candidate-protein findings rather than to establish CAPS or FSTL1 as clinically definitive or hydrocephalus-specific diagnostic biomarkers. Instead, the ELISA results suggest that the expression abnormalities of these candidate proteins retained consistent directionality in the expanded validation samples, providing a stronger rationale for subsequent large-scale, multicenter validation studies and future biomarker-panel development.

Notably, the validation cohort remains relatively small, which limits the robustness and generalizability of the findings. The results primarily support directional consistency rather than diagnostic performance, and formal evaluation of diagnostic metrics, including sensitivity, specificity, and classification performance, was not performed. Therefore, the current findings should not be interpreted as evidence of clinical diagnostic utility.

## 4 Discussion

### 4.1 PHH and iNPH exhibit different exploratory CSF proteomic pattern

The present CSF Olink proteomic analysis suggests that, although PHH and iNPH converge on ventricular enlargement as a shared anatomical phenotype, they show different CSF protein-abundance patterns within this exploratory cohort. Previous studies have established that hydrocephalus is etiologically and pathophysiologically heterogeneous and cannot be reduced to a unitary disturbance of CSF circulation alone (2, 3, 11). In iNPH, both clinical and biomarker studies likewise support the concept that the disorder represents a complex syndrome shaped by altered CSF dynamics, periventricular white matter injury, neurodegenerative change, and local microenvironmental remodeling (12–17). Recent CSF proteomic investigations further indicate that iNPH-related alterations involve synaptic proteins, cell-adhesion molecules, and extracellular-interface networks rather than a single dominant pathway (9, 18, 19). The present findings are consistent with this framework and suggest that PHH and iNPH may be associated with different CSF proteomic contexts despite sharing a similar ventricular phenotype.

Previous CSF protein studies provide a useful external reference for this interpretation: PHH has most often been associated with inflammatory cytokines, blood-breakdown-related injury, extracellular matrix remodeling, coagulation/fibrinolysis, and white-matter injury markers, whereas iNPH studies have more consistently implicated chronic inflammatory mediators, LRG, Alzheimer’s disease-related proteins, synaptic proteins, and cell-adhesion or extracellular-interface molecules (20–24). This contrast is compatible with the view that PHH and iNPH may share CSF-interface involvement but differ in their dominant exploratory annotation context: PHH showed findings more closely aligned with hemorrhage-triggered inflammatory and secretory-interface injury, whereas iNPH showed findings more compatible with chronic neural-connectivity and extracellular-interface annotations (19, 25, 26).

Clinically, the principal relevance of this study does not lie in proposing ready-to-use single-analyte diagnostic assays. Rather, the data identify CAPS and FSTL1 as exploratory candidate proteins that may warrant further evaluation in relation to different hydrocephalus-associated biological contexts. In PHH, CAPS may be associated with CSF-contacting interface reactivity after hemorrhagic injury. In iNPH, FSTL1 may represent an exploratory signal related to chronic microenvironmental remodeling and tissue adaptation. Concomitant differences in WIF1, ARG1, SYT1, NEO1, PLXNA4, CXADR, ITGAV, TNFSF12, and F3 suggest that future studies should evaluate these candidate proteins in relation to broader annotation categories, rather than focusing exclusively on isolated analytes. These results suggest that future biomarker studies in hydrocephalus may benefit from evaluating hemorrhage-associated interface reactivity in PHH and connectivity/interface-remodeling annotations in iNPH as separate exploratory contexts.

### 4.2 CAPS elevation in PHH may be associated with CSF-contacting interface reactivity

The most notable upregulated protein in PHH was CAPS. CAPS is a calcium-binding protein reported in ependymal cells, astrocytes, and subsets of neurons, providing a plausible anatomical link to ventricular and CSF-contacting compartments (27). Experimental studies of PHH have shown that blood breakdown products and innate immune activation can promote choroid plexus activation, inflammatory amplification, and CSF hypersecretion, thereby contributing to ventricular enlargement (5, 28). Within this context, elevated CAPS in PHH is compatible with a hypothesis of calcium-related reactivity at the ventricular-CSF interface, rather than being interpreted as a marker of nonspecific tissue destruction alone. This interpretation is compatible with prior PHH studies showing that CSF inflammatory cytokines, VEGF, and TGF-β-related signals are altered after hemorrhagic ventricular injury; however, the present dataset did not show a simple generalized cytokine-upregulation pattern (21, 22). Instead, CAPS elevation occurred against a background of cytokine-receptor pathway annotation, calcium-signaling representation, cell-adhesion-related protein differences, and lower abundance of neuro-supportive proteins, suggesting that CAPS may be evaluated in future studies as a candidate marker of ventricular-CSF interface reactivity rather than merely acute cytokine release (25).

Its specificity, however, warrants caution. Available evidence does not support CAPS as a hydrocephalus-specific molecule, and its increase may also reflect broader processes, including ependymal stress, ventricular wall perturbation, or more general disturbance of CNS barrier-related interfaces. Even so, in the present dataset CAPS elevation occurred alongside lower abundance of proteins annotated to extracellular regulation, immune homeostasis, and neuro-supportive function, suggesting that it was not an entirely isolated finding within the exploratory protein-abundance pattern.

### 4.3 PHH exhibit lower abundance of proteins annotated to homeostatic and neuro-supportive functions

Compared with CAPS elevation, the more prominent feature of PHH was the coordinated reduction of proteins involved in tissue homeostasis and neural support. WIF1 is particularly informative in this regard. As a canonical extracellular inhibitor of Wnt signaling, WIF1 contributes to the regulation of remodeling programs within the central nervous system (29). Persistent activation of canonical Wnt signaling has been shown to disrupt maintenance of choroid plexus epithelial identity and phenotypic stability (30). Reduced WIF1 in PHH may therefore be compatible with lower abundance of a Wnt-inhibitory protein within the CSF microenvironment. ARG1 is similarly notable. Experimental evidence indicates that depletion of Arg1-positive microglia and macrophages aggravates inflammatory amplification and neural injury, supporting its association with a reparative immune phenotype (31). Reduced ARG1 in PHH may therefore indicate lower abundance of a protein associated with reparative immune phenotypes rather than direct evidence of attenuated inflammation.

This interpretation is reinforced by the broader decrease in molecules related to neuronal integrity and trophic support, including CNTNAP2, CLSTN2, L1CAM, RTN4R, GFRA2, PTPRZ1, VGF, and CCK. Considered together with enrichment of inflammatory signaling, cell-adhesion, lysosomal, and axon-guidance-related annotations, the overall PHH pattern is compatible with possible involvement of CSF-contacting interfaces, lower abundance of extracellular regulatory proteins, and reduced representation of neuro-supportive proteins in the CSF.

The decreased L1CAM signal in the present adult PHH cohort should be interpreted in light of previous neonatal PHH studies reporting increased CSF APP, sAPPα, L1CAM, and related white-matter injury markers (23, 32). Rather than representing a direct contradiction, this difference may reflect variation in age, disease stage, sampling timing, and biological phase: neonatal PHH studies may capture acute injury release and developmental white-matter deformation, whereas the present dataset showed lower relative abundance of neural-supportive and extracellular-interface proteins.

### 4.4 FSTL1 elevation in iNPH may be associated with chronic microenvironmental remodeling

Within the iNPH group, FSTL1 was the most prominent protein requiring individual consideration. FSTL1 is a secreted glycoprotein implicated in development, immune regulation, vascular responses, and tissue remodeling (33). Its role in the nervous system appears context dependent rather than unidirectional. Innate immune activation can induce astrocytic upregulation of FSTL1 (34). In models of cerebral ischemia, FSTL1 has been reported to attenuate apoptosis through the DIP2A/Akt pathway (35), whereas in endothelial systems it has been linked to reduced inflammation and modulation of transcellular transport (36). Against this background, increased CSF FSTL1 in iNPH is most appropriately interpreted as an exploratory candidate signal related to chronic low-grade inflammation, interface adaptation, and tissue remodeling rather than as a simple marker of either injury or compensation. This interpretation is concordant with previous iNPH CSF studies showing increased LRG and TGF-β-related signaling, together with more recent evidence for inflammatory-protein alterations involving MCP-1, CCL4, and PD-L1 (24, 26, 37). Within this context, FSTL1 is best positioned as an exploratory candidate protein within a chronic inflammation, vascular-interface, and extracellular-remodeling annotation context, rather than as an isolated iNPH-specific biomarker.

### 4.5 Reduced abundance of neural-connectivity and interface-homeostasis proteins was a prominent finding in iNPH

Relative to the limited number of upregulated proteins, a prominent observation in iNPH was the concurrent lower abundance of molecules annotated to neural connectivity and interface stability. SYT1 is a presynaptic vesicle protein, and CSF synaptotagmin-1 has previously been used as an indicator of synaptic dysfunction (38). NEO1 should not be viewed solely as a developmental marker, as neogenin-1 also participates in trans-synaptic signaling and contributes to long-term potentiation in the dentate gyrus (39). PLXNA4 is a key component of the semaphorin-plexin axon-guidance system and directly regulates nerve-fiber guidance (40). The concurrent lower abundance of SYT1, NEO1, and PLXNA4 is therefore consistent with reduced representation of proteins associated with synaptic maintenance, neural connectivity, and axon-guidance-related signaling in iNPH. This interpretation is in keeping with recent large-scale CSF proteomic studies demonstrating reduced synaptic and adhesion-related proteins in iNPH (19).

This observation is further supported by recent unbiased and outcome-oriented CSF proteomic studies in iNPH, which identified molecular signatures involving synaptic organization, cell–cell adhesion, extracellular matrix organization, inflammatory signaling, and axon-development-related proteins (19, 41, 42). The concordance between these external proteomic datasets and the present lower abundance of SYT1, NEO1, PLXNA4, SLITRK-family proteins, C1QL2, CBLN4, CXADR, and ITGAV supports the interpretation that reduced abundance of proteins annotated to neural connectivity and interface homeostasis is a prominent observation in the present iNPH CSF dataset.

Proteins annotated to interface homeostasis were likewise reduced. CXADR is a tight-junction-associated transmembrane protein involved in epithelial permeability and tissue homeostasis (43, 44). Its reduction may indicate diminished stability of intercellular interfaces, although it is not sufficient on its own to support classification as a specific marker of blood–brain barrier injury. ITGAV is closely linked to cell-matrix adhesion, TGF-β activation, and interface signaling (45). Reduced ITGAV therefore supports further evaluation of matrix-interface communication as a candidate annotation context. TNFSF12 encodes TWEAK, and the TWEAK/Fn14 axis has established roles in CNS inflammation, vascular permeability, and injury responses, including modulation of inflammatory properties in human blood–brain barrier endothelial models (46, 47). Reduced TNFSF12 in iNPH may therefore represent lower abundance of a protein associated with chronic inflammatory and interface-related signaling contexts. F3 is best considered here as a molecule situated at the intersection of coagulation, vascular signaling, and inflammatory interface biology. Although tissue factor has recognized roles in CNS hemostasis and neuroinflammatory disease (48), its specific significance in iNPH remains to be defined.

### 4.6 Cilium- and basal body-related annotations may inform hypotheses about interface remodeling in iNPH

Reactome analysis additionally identified enrichment of pathways related to cilium assembly, basal body anchoring, and centrosome maturation. Previous studies support a close relationship between ependymal cilia, local CSF flow, and ventricular homeostasis (49). At the same time, ciliary dysfunction should not be invoked as a universal or sufficient explanation for all forms of human hydrocephalus (50). The present data therefore support a more restrained interpretation, namely that the iNPH-associated DEP list showed enrichment of annotations related to ependymal surface, basal body, and microtubule-associated categories, but the available evidence is insufficient to classify iNPH as a canonical ciliopathy.

### 4.7 Candidate protein findings may inform barrier-oriented therapeutic route selection

From the perspective of CNS barrier-oriented drug delivery, the candidate proteins identified here may be more informative for biologically prioritizing therapeutic entry interfaces than for immediate diagnostic application. In this context, the key translational question is which disease-relevant CNS interface is most strongly suggested by the exploratory proteomic annotations in each hydrocephalus subtype and may therefore represent a candidate compartment for therapeutic targeting and response monitoring in future studies (3, 51).

In PHH, the translational interest of CAPS lies primarily in its association with the ventricular-ependymal/choroid plexus-blood-CSF barrier interface. Experimental studies have shown that inflammatory activation and secretory reprogramming of the choroid plexus are central components of PHH pathophysiology, including TLR4-NF-κB-SPAK-NKCC1-driven cerebrospinal fluid hypersecretion (3, 5, 28, 52). This interface is also therapeutically actionable in preclinical systems: inhibition of the relevant secretory pathway can reduce CSF hypersecretion, choroid plexus-targeted NKCC1 modulation can enhance CSF clearance and attenuate ventriculomegaly, and more recent blood-CSF barrier-oriented nanodelivery strategies have shown proof-of-concept benefit in restoring barrier integrity while suppressing hypersecretion (5, 28, 51, 53). In this setting, CAPS should not be interpreted as evidence of a transport mechanism per se; rather, it is compatible with the hypothesis that PHH-associated proteomic differences may be weighted toward the ventricular-CSF interface. Its potential translational value would need to be tested in future studies evaluating choroid plexus- or ventricular interface-directed interventions and candidate pharmacodynamic readouts.

In iNPH, by contrast, increased FSTL1 may be compatible with a chronic remodeling-prone microenvironment involving low-grade inflammatory activity, extracellular-interface alteration, and vascular or endothelial adaptation rather than overt secretory barrier activation. Current biomarker studies in iNPH do not support a single disease-defining CSF signature, but they increasingly implicate inflammatory signaling, extracellular matrix-related changes, and broader microenvironmental remodeling in disease biology (8, 54, 55). Within this framework, the relevance of FSTL1 to drug delivery is better understood in terms of route stratification than target specificity. Notably, outside hydrocephalus, FSTL1 has been linked to endothelial regulation, including reduced transcytosis and attenuated inflammatory activation (36). Accordingly, future studies may need to evaluate whether trans-barrier delivery, barrier-modulating strategies, or CSF-route administration is biologically better aligned with the intended target compartment when exploratory annotations suggest endothelial-matrix remodeling rather than pronounced CSF hypersecretion (36, 51). The potential translational value of FSTL1 would be as a candidate readout for interventions directed at endothelial homeostasis, chronic inflammation, or extracellular-interface remodeling, pending validation in larger cohorts (51).

Collectively, the relevance of CAPS and FSTL1 to drug delivery lies in their potential to inform prioritization of therapeutic entry sites, route selection, and treatment-response monitoring rather than to establish the superiority of any specific delivery strategy. However, the present study did not directly evaluate BBB or blood-CSF barrier permeability, intracranial drug distribution, or transport efficiency. These implications should therefore be regarded as translational inferences and future directions rather than direct functional conclusions.

### 4.8 Limitations

The present findings should be interpreted in light of several important limitations. Most importantly, the sample size was small, and the study was designed as an exploratory discovery-stage analysis rather than a diagnostic or clinically definitive investigation. Under these conditions, CAPS and FSTL1 cannot be regarded as hydrocephalus-specific biomarkers. Within the current cohort, no stable or clinically informative relationships were identified between these proteins and patient clinical presentation, imaging characteristics, or prognosis. At the same time, subsequent ELISA testing in an expanded patient set still showed clear differences in protein expression between hydrocephalus cases and controls. This provides preliminary orthogonal support for the direction of the Olink findings, but it does not remove the need for larger independent validation cohorts.

The PHH cohort was also assembled under specific inclusion and exclusion criteria and therefore may not fully represent the broader clinical spectrum of PHH. Selection bias must therefore be considered when interpreting the observed CSF proteomic patterns. Differences in hemorrhage subtype, disease stage, treatment exposure, and associated tissue injury may all influence the molecular profile and may limit the generalizability of the present results.

In addition, potentially relevant clinical comorbidities were not controlled in a formal manner. Diabetes mellitus, hypertension, and vascular disease may affect CSF protein composition, particularly in disorders accompanied by chronic vascular remodeling and barrier-interface disturbance. Future studies will require more rigorous sample selection and more carefully stratified grouping strategies in order to minimize confounding and isolate disease-related molecular signals as clearly as possible.

Another limitation concerns the non-hydrocephalus control group. The non-hydrocephalus control group CSF samples were obtained from patients with primary hemifacial spasm or primary trigeminal neuralgia undergoing elective surgery, rather than from completely healthy volunteers. These disorders are usually focal cranial nerve conditions related to neurovascular compression and are not typically associated with ventriculomegaly, intracranial hemorrhage, CNS infection, tumor, or diffuse neuroinflammatory or neurodegenerative disease. Their influence on the global CSF proteomic profile is expected to be relatively limited, but they cannot be considered strictly healthy controls. The non-hydrocephalus control group is not derived from a healthy general population. Due to limitations in clinical assessment, underlying comorbidities may still have influenced the composition of cerebrospinal fluid. Future studies should include larger, better-matched control cohorts.

Multiple testing should also be considered. In this exploratory Olink proteomic analysis, differential protein expression was initially screened using nominal *p* values, while Benjamini–Hochberg false discovery rate (FDR)-adjusted p values (q values) were calculated. In the PHH versus the non-hydrocephalus control group comparison, a total of 85 proteins remained statistically significant after FDR correction. In contrast, no proteins reached statistical significance after FDR correction in the iNPH group (all q > 0.05). Among representative findings, CAPS remained significant after FDR correction in the PHH group, whereas FSTL1 did not retain significance in the iNPH group. To enhance sensitivity in this small-sample exploratory study, protein selection for downstream analyses was primarily based on nominal p value thresholds. Therefore, the results should be interpreted as preliminary and hypothesis-generating rather than confirmatory. Before any clinically meaningful biomarker or subtype-specific molecular conclusions can be drawn, further validation in larger, independent, multicenter, and longitudinal cohorts is required.

## Data Availability

The original contributions presented in the study are publicly available. These data can be found here: Figshare, DOI: 10.6084/m9.figshare.32091790. The raw Olink NPX data, clinical information, and ELISA validation data are also provided in the Supplementary material.

## Supplementary material

Supplementary Data Sheet 1 Raw Olink NPX data.

Supplementary Table 1 ELISA validation data for CAPS and FSTL1.

Supplementary Table 2 Clinical information.
